# Variation in handshape and orientation in British Sign Language: The case of the ‘1’ hand configuration

**DOI:** 10.1016/j.langcom.2012.09.001

**Published:** 2013-01

**Authors:** Jordan Fenlon, Adam Schembri, Ramas Rentelis, Kearsy Cormier

**Affiliations:** a Deafness, Cognition & Language Research Centre, University College London, UK; b La Trobe University, Melbourne, Australia

**Keywords:** Sign language, Sociolinguistic variation, Phonology, Pointing

## Abstract

This paper investigates phonological variation in British Sign Language (BSL) signs produced with a ‘1’ hand configuration in citation form. Multivariate analyses of 2084 tokens reveals that handshape variation in these signs is constrained by linguistic factors (e.g., the preceding and following phonological environment, grammatical category, indexicality, lexical frequency). The only significant social factor was region. For the subset of signs where orientation was also investigated, only grammatical function was important (the surrounding phonological environment and social factors were not significant). The implications for an understanding of pointing signs in signed languages are discussed.

## Highlights

► We study sociolinguistic variation at the phonological level in British Sign Language. ► Of the linguistic factors, the surrounding phonological environment is most important. ► Of all the social factors considered, only region is important. ► The orientation of pointing signs is constrained by grammatical function.

## 1 Introduction

In this paper, findings from the first major study on phonological variation in British Sign Language (BSL) are presented. Using data collected as part of the BSL Corpus Project (Schembri et al., 2011), we examine sociolinguistic variation in signs with the ‘1’ handshape (i.e., with an extended index finger), using methodology adapted from work by Bayley et al. (2002) on American Sign Language (ASL). In their study, Bayley et al. found that the most important factor influencing this phonological variation was grammatical category but that a number of other factors, including features of the immediate phonological environment and social factors (e.g., age, ethnicity, language background, and region) played a role. For the current study, we investigate if the same factors influencing 1 handshape variation in ASL are also at work in an unrelated sign language and whether other factors not considered by Bayley et al., such as lexical frequency or indexicality, might also be involved. We use a variationist approach to investigate both the social and linguistic factors that may be relevant in conditioning variation (e.g., Walker, 2010). Additionally, since we are particularly interested in the pointing signs in our data, we have further examined all pronominal and locative pointing signs for variation in orientation and possible conditioning linguistic and social factors.

In the following sections of the paper, we first provide some background about sociolinguistic variation in BSL, before discussing studies of phonological variation in sign languages generally. Next, we present a summary of previous work on 1 handshape variation in ASL, before discussing the BSL Corpus Project data and our studies into handshape and orientation variation. Lastly, we discuss the implications of our work for an understanding of phonological variation in sign languages and its relationship to gesture, lexical frequency and models of the sign language lexicon.

## 2 Background

### 2.1 Sociolinguistic variation and change in BSL

Research conducted into sociolinguistic variation in BSL has concentrated almost exclusively on the lexical level. For example, it is well documented that there is considerable regional variation in the BSL lexicon, with traditional signs for colour terms and numerals, for example, often being specific to a region in the UK (Brien, 1992; Deuchar, 1984; Sutton-Spence and Woll, 1999; Sutton-Spence et al., 1990). More recent research has, however, revealed that the use of traditional regional signs for countries, numbers and colours, appears to be diminishing (Stamp et al., in press). Stamp et al. suggest that these linguistic changes reflect social changes in the deaf community and in the use of communications technology, with greater exposure to sign variants on the internet and television and deaf people generally being more mobile, both nationally and internationally, than in the past.

To date, the largest study of sociolinguistic variation in BSL focuses on fingerspelling (i.e., the use of a two-handed manual alphabet to spell out English lexical items, a system used to enable the borrowing of words from the surrounding spoken language) (Sutton-Spence et al., 1990). Sutton-Spence et al. used a dataset of 19,450 fingerspelled items collected from 485 interviews with BSL signers on the British deaf television programme *See Hear*. They found age and region to be significant factors in predicting use of the two-handed manual alphabet with younger signers and signers from the south-western region of England producing the least fingerspelling.

No large-scale studies have as yet focused on variation in BSL at the phonological level. The research conducted here is thus the first attempt to investigate phonological variation in BSL and its possible relationship to linguistic and social factors.

### 2.2 Sign language phonology

**Fig. 1:**
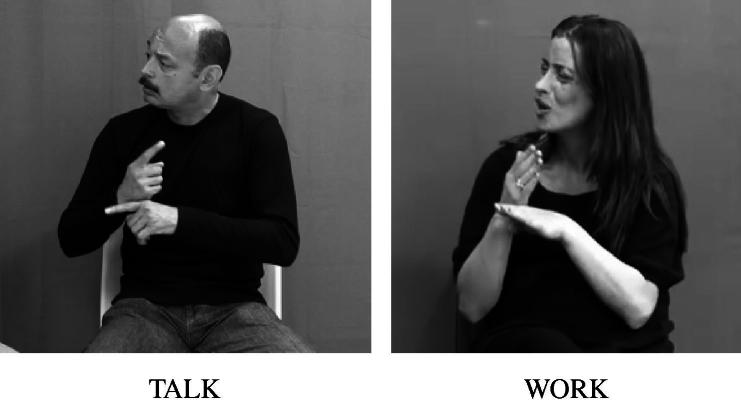
A minimal pair in BSL.

William Stokoe (1960) was the first to propose that the manual signs in sign languages could be analysed into three main sublexical elements: handshape, location, and movement. These contrastive components may be considered analogous to the parameters of speech production, such as voicing, place and manner of articulation. BSL signs are made from the combination of a limited set of parameter values (for example, there appear to be around 35 distinctive handshapes in the language), and minimal pairs may be distinguished on the basis of differences in these parameters (Brennan et al., 1984). For example, the BSL signs talk^1^ and work have the same location (i.e., they are articulated on the radial side of the non-dominant hand in neutral space) and the same movement (i.e., a repeated tapping movement) but differ in handshape (i.e., talk is produced with the 1 handshape whilst work is produced with the B handshape) as shown in Fig. 1 below.

Although there is debate about the relationship between these three parameters and additional formational elements (such as the orientation of the hands, see Sandler and Lillo-Martin, 2006) and about the nature of minimal pairs in sign languages (Liddell and Johnson, 1989), evidence for the sublexical compositionality of signs is well-established. This includes notions of well-formedness of signs shared by native signers and documented in dictionaries (e.g., Brien, 1992 for BSL), the relevance of these parameters for sign language processing (e.g., Orfanidou et al., 2010), and stages of phonological development in sign language acquisition (e.g., Mann et al., 2010). Since the 1970s, phonologists have also begun to propose models in which the parameters of handshape, location and movement can be represented as bundles of distinctive features (e.g., Brentari, 1998; Crasborn, 2001; Sandler, 1989). Handshapes, for example, can be analysed as in terms of the behaviour of (a) the thumb: whether it is extended away from the rest of the hand, for example, or held across the other fingers, and (b) the fingers: which of the fingers are selected (i.e., actively involved in the articulation of a sign or not) and whether they are spread apart or together, bent at specific joints or fully extended.

### 2.3 Overview of phonological variation and change in signed languages

Although there have been a number of studies on phonological variation in signed languages, many of these studies are small both in number and scale when compared to studies on phonological variation in spoken languages. Furthermore, since the first study published by Battison (1974), work on phonological variation has focused almost exclusively on ASL. It is only recently that studies on phonological variation in other sign languages have begun to be undertaken, including work on Australian Sign Language (Auslan) and New Zealand Sign Language (NZSL) (Schembri et al., 2009). Together, these studies begin to provide us with a cross-linguistic perspective on phonological variation in sign languages.

Previous investigations have underlined the influence of linguistic and social factors in sign language phonological variation. For example, Battison (1974) collected data on the deletion of the subordinate hand in two-handed signs (known as ‘weak drop’ see Brentari, 1998) and found that this process was more common in symmetrical than asymmetrical two-handed signs. Bayley et al. (2002) describe several studies (dating back to the 1970s) examining phonological variation in ASL such as Woodward et al.’s (1976) investigation into the variable use of ASL signs that have related forms produced on the face or on the hands. Drawing on questionnaire data from 45 participants, these researchers found evidence of variation due to ethnicity, with black signers much more likely to use the hand rather than face variants. Their data also suggested regional differences, with signers in New Orleans producing fewer variants on the hands than those in Atlanta. Another study by Woodward and DeSantis (1977) found ethnic variation in two-handed versus one-handed forms of some ASL signs, with white signers using significantly more of the one-handed variants of these signs. They also found that signers from the southern states of the US used more two-handed variants than non-southerners, and that older signers used more than younger signers.

Later, accounts of phonological variation in signed languages emphasised the key role that the immediate phonological environment plays in this variation with respect to all three major parameters (e.g., Liddell and Johnson, 1989; Johnston, 1989). That is, phonological features of a given sign, such as handshape and location, are likely to assimilate to the features of neighbouring signs, causing variation away from the citation form (i.e., the form which is produced in isolation and listed in dictionaries). Although the importance of the immediate phonological environment for phonological variation has been given empirical weight in recent analyses using large corpora which have focussed on 1 handshape variation (e.g., Bayley et al., 2000, 2002) and variation in signs produced at the forehead location (e.g., Lucas et al., 2002; Schembri et al., 2009), these studies demonstrate that other factors in addition to the phonological environment may be at work in conditioning variation. For example, depending on the target variable, some grammatical categories of signs (e.g., pronouns or verbs) can be expected to vary more compared to others. Schembri et al. (2009), in applying the work of Lucas et al. (2002) to Auslan and NZSL data, initially found grammatical category to be the most important factor but discovered an interaction between location variation and lexical frequency. Highly frequent verbs were found to occur significantly more frequently in lowered form (i.e., at locations lower than the forehead) than all other sign types (e.g., high frequency nouns and adjectives and low frequency nouns, adjectives and verbs). This observation about the relationship between lexical frequency and phonological variation runs parallel with findings in spoken languages (Bybee, 2002; Philips, 1984).

In addition to linguistic factors, the studies mentioned above have demonstrated that social factors have an important role in phonological variation in signed languages. Social factors found to be relevant include region, age, gender, language background (i.e., whether a participant was raised in a deaf signing family or not), ethnicity, and social class. These social factors and the extent to which they influence phonological variation, however, appear to vary across sign languages. For example, region and gender were found to be important factors in location variation for the Auslan and NZSL location studies. Age, however, was only a significant factor in Auslan, and language background and ethnicity were found to be significant in NZSL only. Furthermore, although the NZSL and ASL studies of location variation both found language background and ethnicity were important, their results indicated different effects in each community (e.g., native signers used significantly more citation forms in ASL while native signers tended to use more variant forms in NZSL).

Similar observations reported in the studies above have been made for BSL but on the basis of relatively little data. Deuchar (1981) claimed that the deletion of the non-dominant hand in symmetrical two-handed signs, such as give and hospital, was frequent, as also noted in ASL (Battison, 1974). Deuchar argued that this weak drop in asymmetrical two-handed signs appeared most likely in signs where the handshape on the non-dominant hand was a relatively unmarked configuration, such as the B handshape (see illustration of this handshape in Table 2). Thus, variants without the non-dominant hand seemed more common in her data in signs such as right (with a non-dominant B handshape) than in father (non-dominant H; see Table 2 for illustration). In a small pilot study, Deuchar also found that discourse factors underlie the frequency of this phenomenon with informal varieties producing more tokens of weak drop than formal varieties. Deuchar (1984) later suggested that this weak drop variation may also reflect language change in progress, based on Woll’s (1981) claim that certain signs (e.g., again) which appear to be now primarily one-handed in modern BSL were formerly two-handed. Furthermore, Deuchar (1984) notes that progressive and regressive assimilation of handshape is possible in BSL and suggests that there may be underlying grammatical constraints at work. However, a thorough analysis of how such phonological processes work in BSL has yet to be undertaken.

Despite considerable work on the theoretical aspects of phonology in the field of sign language linguistics (e.g., Brentari, 1998; Crasborn, 2001; Sandler and Lillo-Martin, 2006), there remain relatively few studies examining sociolinguistic variation at the phonological level in signed languages. A thorough cross-linguistic perspective on sociolinguistic variation and change in phonology is needed, however, so that we can build a better understanding of what kind of internal and external factors constrain phonological variation in signed languages, and how these factors compare to those found in spoken languages.

### 2.4 Variation in the 1 handshape

In an influential paper on ASL phonology, Liddell and Johnson (1989:250) made a specific claim about signs with the 1 handshape. They observed that the hand configuration of the first person pronominal sign in ASL (which they referred to as me; the form of this sign is identical to pt:pro1 that we report here for BSL) ‘typically assimilates to that of a contiguous predicate in the same clause’. They went on to claim that ‘the extent to which signs other than me assimilate to the hand configuration of another sign, although not yet thoroughly investigated, appears to be considerably more limited.’

This claim was investigated for the first time by a large-scale study examining variation in the 1 handshape in ASL conducted by Bayley et al. (2002). They collected 5356 sign tokens from 207 signers engaging in spontaneous conversation (filmed in groups or in pairs). Their pool of participants consisted of native or near native signers (those who had learnt to sign before the age of 6) in a quota sample mixed for gender, age, language background, ethnicity and social class and recruited from seven sites around the United States: Staunton, Virginia; Frederick, Maryland; Boston, Massachusetts; Olathe, Kansas; New Orleans, Louisiana; Fremont, California; and Bellingham, Washington. An analysis of 5195 tokens, excluding the thumb-only variant,^2^ revealed that signs that used the 1 handshape in citation form were more often realised with some other handshape (60% of their data). This suggested that this variation was not as limited an occurrence as previously claimed by Liddell and Johnson (1989), although the first person pronoun did indeed account for a considerable proportion of this variation. Despite this large amount of variation, almost all of the variant tokens could be grouped into just two main handshape categories: the L handshape (30%), and the 5 handshape (25%). The remaining 5% of their data was divided between two other variants: those with the 4 handshape and the X handshape (see Table 2 for illustrations of these handshapes). Each token was analysed for a number of linguistic and social factors. Results indicated that grammatical category was the most important factor conditioning variation in the 1 handshape in ASL. Specifically, nouns, adjectives, verbs, adverbs, wh-signs, and third person pronouns (singular and plural forms of all pronouns were not distinguished in the analysis) appeared significantly more frequently in citation form. Conversely, first and second person pronouns tended to appear more often in non-citation form (i.e., using some handshape other than the 1 handshape). This finding led Bayley et al. to conclude that there is a systematic correspondence between indexicality and the degree of variation observed in handshape. Specifically, Bayley et al. argued that first person pronouns, the category that was found to vary most over others, is also the category considered to be the most indexic (both singular and plural forms involve points towards the chest of the signer). This is followed by second person pronouns, the next category found to vary the most, which they analysed as less indexic than first person pronouns but more so than third person pronouns (typically, the singular and plural forms both involve a point towards the signer’s conversational partner(s)). In contrast, third person pronouns, signified by a point that indicates a non-addressed referent or group of referents (whether physically present or not), Bayley et al. propose, are the least indexic of the three and tend to favour citation form in the ASL data. Finally, Bayley et al. note that in non-indexical grammatical and content signs, the handshape has more semantic relevance and therefore is likely to retain its citation form. Additionally, whilst all categories of non-pronominal signs were observed to vary in handshape, they were less likely to show the same degree of handshape assimilation as pronouns (i.e., there was assimilation in fewer of the distinctive features in the handshape of signs in these other categories) since doing so may have a relatively greater effect on its perceived meaning (i.e., the sign may be interpreted as a different sign by the conversational partner).

Bayley et al. (2002) also found features of the immediate phonological environment to have a significant effect on 1 handshape variation. In many tokens, handshape features of the target sign appeared to show assimilation to features of the signs before and after the target sign. That is, if the handshape before was articulated with an extended thumb (i.e., open and extended to the side of the hand), it was likely to observe a variant with an extended thumb in the target sign. Conversely, if the handshape before was articulated with an opposed thumb (i.e., a thumb held across the fingers), it was more likely to observe an opposed thumb variant in the target sign. Bayley et al. found a clear relationship between the number of features the citation form of the target sign shares with adjacent signs and the likelihood that it will retain its citation form. An analysis of this finding alongside grammatical category suggests that the two categories are working in tandem. That is, when more features are shared with the target sign as a noun it is more likely to keep its citation form than when it is a first person pronoun.

In addition to linguistic factors, Bayley et al. found a range of social factors to be significant factors conditioning variation in the 1 handshape in ASL: region, age, language background, social class and ethnicity. The remaining social factor in their analysis, gender, was not found to be significant. Results by region tended to fall into two groups. Signers from California, Kansas/Missouri, Louisiana and Massachusetts all used significantly more citation forms, whilst signers from Maryland, Virginia and Washington used fewer citation forms. Within this regional grouping, further interaction with the remaining significant social factors was observed. Younger signers from California, Kansas/Missouri, Louisiana and Massachusetts used significantly more citation forms, for example, whereas older signers did not (although this was not a strong effect). Similarly, middle class signers in Maryland, Virginia and Washington used more citation forms than working class signers. In contrast, working class signers in California, Kansas/Missouri, Louisiana and Massachusetts produced more examples of the citation form than middle class signers. The results for language background as a significant factor were mixed. When a specific handshape variant was selected as the dependent variable (the 5 handshape variant in which all fingers and the thumb were extended), signers with deaf signing parents in California, Kansas/Missouri, Louisiana, and Massachusetts were found to use significantly fewer tokens of this variant, whilst signers with hearing parents in the same areas used more. Language background was not found to be significant in analysis restricted to Maryland, Virginia and Washington alone. Finally, ethnicity was found to be a significant factor influencing variation in the 1 handshape: black Americans used significantly more examples of the citation form compared to white Americans. However, the authors conclude that this result is a reflection of regional differences in preference for citation form rather than ethnicity itself. That is, the number of African Americans in areas that tended not to use citation forms (e.g., Maryland, Virginia and Washington) was so low that a meaningful analysis based on ethnicity could not be achieved.

Although sign language phonologists had observed these patterns previously (e.g., Corina, 1990; Liddell and Johnson, 1989; Sandler, 1999), Bayley et al. (2002) was the first study to provide empirical evidence from a large naturalistic dataset for handshape assimilation effects in any signed language. Furthermore, it was one of the first to show that grammatical function appeared to be a significant factor conditioning the frequency of variant forms. The work of Bayley et al., however, did not include any investigation into the possible effect of individual lexical items on the data. For example, although the results for specific person forms for pronouns are presented, singular and plural forms of the first person pronoun are grouped together. Guy (2007) points out that research on phonological variation in spoken languages has shown that phonological variation does not affect all lexical items sharing particular phonological features in the same way (for example, final stop deletion in the English word *and* is much more frequent than other words with a word final stop). One of the key factors related to these lexical effects in phonological variation is lexical frequency. Bayley et al. did not investigate frequency effects in their data, despite the fact that singular and plural forms of the first person pronoun represented almost 40% of their entire dataset. Furthermore, they claim that there is a relationship between indexicality and variation in the first, second and third person forms. In order to strengthen this claim, there is a need to separate out plural and singular forms of pronominal signs, as there is some evidence that plural pronoun forms are less indexic than singular forms in both ASL and BSL (Cormier, 2007).

With regard to the influence of social factors, Bayley et al. (2002) identified significantly different degrees of handshape variation in different regional groupings due to social class and age. Despite this, Bayley et al. (p. 49) stressed the “near uniformity of constraint effects across regional and social groups” that illustrated that ASL users constituted a single language community. They suggested that differences between some social subgroups in the patterns of 1 handshape variation in first person and non-first person pronouns (with significantly greater handshape assimilation in the first compared to non-first person pronominals) reflects the possibility that grammaticalisation of a first versus non-first person distinction is at work, and that it is unfolding at different rates in different subsets of the American deaf community.^3^ An issue not considered by Bayley et al., however, is the degree to which such variation also occurs in pointing gestures, particularly between pointing gestures to the self and to others. An analysis of 388 body-directed gestures by Cooperrider (2011), collected from a set of televised interviews with 40 native speakers of American English, indicated that only 10% of ‘self-points’ used a handshape with an extended index finger (with or without thumb extension). The figure in the ASL data was different, with about 48% of all first person pronouns using the 1 handshape (thumb opposed), or the related L handshape (thumb extended). Handshape variants other than a 1 or L handshape accounted for 52% of the ASL data, but almost 90% of the gesture data. Unfortunately, however, Cooperrider did not undertake studies of handshape assimilation effects in his gesture data, nor do we have comparable figures for pointing gestures to non-first person referents. Nevertheless, the grammaticalisation hypothesis needs to take into account the patterns of variation in related pointing gestures used by non-signers which are undoubtedly related to these forms in sign languages, both in form and function (Cormier et al., submitted for publication; Johnston, in press).

### 2.5 Variation in orientation in pointing signs

A large proportion of the signs with a 1 handshape that appear in sign language discourse involve pointing signs of various types (Johnston, 2012; McKee and Kennedy, 2006; Morford and MacFarlane, 2003). This appears to be especially true of the spontaneous conversational BSL data used in this study (Cormier et al., 2011). A number of researchers have suggested that variation in the orientation of pointing signs is conditioned by the grammatical function of the pointing sign. Pfau (2010) provides an overview of pointing in sign languages, showing how a number of researchers working on a range of sign languages (de Vos, 2008; Engberg-Pederson, 2003; van der Kooij et al., 2006) have proposed that pronominal or anaphoric pointing tends to occur with a palm vertical orientation, whereas locative pointing favours palm down orientation. To our knowledge, there has been no empirical investigation of these claims (certainly not with a large dataset), nor has the relationship to similar patterns in co-speech pointing gestures been explored (similar claims about the relationship between different indexical functions and orientation has been made for Neapolitan gesture, see Kendon and Versante, 2003).

## 3 Research questions

We had two main research questions that motivated our study of sociolinguistic variation and change in the 1 hand configuration in BSL. First, we wanted to know what conditions handshape variation in signs that have the 1 handshape in citation form in BSL compared to ASL, given that the two sign languages are historically unrelated. Would the same linguistic and social factors be involved? Would additional factors, such as lexical frequency, be important? Second, we were interested in understanding orientation variation in signs with the 1 handshape in BSL: Which linguistic and/or social factors would condition orientation variation in BSL? Also, would the analysis reveal the kind of systematic difference between pronominal and locative pointing signs that has been suggested in the sign language literature for other sign languages?

## 4 Methodology

### 4.1 BSL Corpus Project

The study reported in this paper draws on data collected as part of the BSL Corpus Project (Schembri et al., 2011), a large-scale project that aimed to create the first online, open-access corpus of BSL. The BSL Corpus features digital video data collected from 249 deaf signers from eight urban centres around the United Kingdom. All participants were filmed while engaged in four tasks: retelling a personal experience narrative, participating in a 30-min conversation, answering questions on language attitudes and awareness, and responding to a short lexical elicitation task. The large, semi-stratified sample and the type of data collected makes the BSL Corpus dataset an ideal resource for the questions put forward in this study. For this study, we focussed on data from 211 signers across 7 cities. The distribution of participants according to several social categories is provided in Table 1. In the following sections, the BSL Corpus Project methodology in regard to these categories is described in more detail.

#### 4.1.1 Sites

Phonological variation due to region is a widely-studied phenomenon in British English (for an overview, see Trudgill (1999)). We planned to include region as a social factor in our analysis of 1 handshape variation because results from previous studies in phonological variation in other signed languages (e.g., Bayley et al., 2002; Lucas et al., 2002; Schembri et al., 2009) have suggested that region is an important factor in conditioning variation in signed languages as well. For this study, we included data from 7 cities representing some of the major urban centres in the four regions of the United Kingdom: Birmingham (in central England), Bristol (in the southwest), London (in the southeast), and Manchester (in the northwest); Belfast in Northern Ireland; Glasgow in Scotland; and Cardiff in Wales. To ensure that each participant was representative of their region, only participants who had lived or worked in that region for ten years or more were invited to take part in the BSL Corpus Project.

#### 4.1.2 Participants

A total of 211 deaf participants were included in the study. In terms of language background, 94% (*n* = 199) of participants included in this study reported learning to sign before the age of 7 and all the remaining individuals reported that they learnt to sign by the age of 12. Native signers (i.e., those from a deaf family exposed to BSL from birth) represented 32% (*n* = 68) of participants. Research has shown that the age at which a child is exposed to sign language as a first language has a considerable effect on their sign language proficiency in adulthood (for a review, see Emmorey, 2002).

The participant sample was balanced for gender. Of the 211 participants, 52% (*n* = 109) were women. It is well documented that gender is a significant factor in phonological variation in spoken languages (e.g., Cheshire, 2002). This has also been found for signed languages (e.g., Lucas et al., 2002; Schembri et al., 2009).

Age-related variation is well documented for spoken languages (e.g., Bailey, 2002) and for signed languages, including BSL (Lucas et al., 2002; Stamp et al., in press; Sutton-Spence et al., 1990). Due to variable patterns of language transmission within the deaf community (the language is sometimes learned from peers in schools for deaf children, or after school as a young adult in other settings), clear differences can be seen between older and younger signers (for example, in the use of lexical items, as explained above, see Sutton-Spence et al., 1990; Stamp et al., in press). Recruitment to the BSL Corpus Project was designed to reflect this variation by ensuring that participant selection was spread across age groups, ranging from 16 to 94 years of age. For the study reported here, participants were grouped into two categories according to age: younger (16–50) and older (50–94). The division of participants into these age groups is partly motivated by changes in language policy in deaf education during the twentieth century (e.g., from education that emphasised the exclusive acquisition of speech and listening skills to increasing acceptance of sign language in the classroom; see Woll and Ladd (2011) for an overview).

Phonological variation due to social class is well-established in spoken languages (e.g., Ash, 2002), and there is some evidence for it being a relevant social factor for phonological variation in ASL, although often in interaction with other social factors. It was found to be relevant for an understanding of 1 handshape variation in ASL, although in this case (as described above), it exhibited a complex interaction with region (Bayley et al., 2002). For the current study, we classified participants into two broad social classes based on their occupation and/or educational background. Deaf individuals with a university education and/or ‘white collar’ professional occupations were categorised as middle class, whereas individuals with no university education and having traditional ‘blue-collar’ factory or trade-related occupations were classified as ‘working class’.

Ethnicity is an important social factor in sociolinguistic variation in many English-speaking communities (Fought, 2002), and has been shown to be relevant to studies of phonological variation in ASL (Lucas et al., 2002). The ethnic composition of the British deaf community is, however, unknown. As the overall British population was reported to be about 10% non-white (mostly of Afro-Caribbean and south Asian origin) in the 2001 Census (Office for National Statistics, 2006), we attempted to include a similar proportion of non-white participants in our project.

#### 4.1.3 Data collection

Data collected as part of the BSL Corpus Project involved working with a deaf fieldworker from each site who conducted participant recruitment (a methodology first used in deaf communities by Ceil Lucas and colleagues, see Lucas et al., 2001). All fieldworkers were deaf and all but one were native signers. All fieldworkers were also present on the day of filming and assisted in data collection together with a deaf project researcher. No hearing people were present during filming. This kept possible language contact influences to a minimum (Lucas and Valli, 1992). Participants were always filmed in pairs and, where possible, were filmed with another person within, or close to, their age group. As participants were required to hold a conversation for 30 min, they were partnered with someone familiar (i.e., a friend) so that they felt relaxed and any awkwardness could be avoided. Filming sessions took place in settings familiar to the participants, e.g., deaf centres and deaf organisations, to ensure that participants felt comfortable and that it was appropriate to use a relatively informal variety of BSL.

#### 4.1.4 Data coding

For the 1 handshape variation study, we coded 10 sign tokens each from 211 participants participating in the first, more informal half of the filming session, which included participants recounting personal narratives and/or engaging in free conversation. These sign tokens were signs that were known to have the 1 handshape in their citation form. These tokens include pronominal pointing signs (i.e., first, second, and third person pronouns), locative pointing signs (e.g., pt:loc meaning ‘there’ or ‘here’), wh-signs (e.g., what, who, why), conjunctions (e.g., but, and) as well as nouns (e.g., information, england, girl), verbs (e.g., talk, debate, say) and adjectives (e.g., quick, slow).

All the lexical signs in this study were assigned an ID gloss, which represents emerging best practice when annotating a sign language corpus (Johnston, 2010). An ID gloss is a unique label used to identify a particular lexeme and to represent all its phonological and morphological variants in the process of lemmatisation. ID glosses do not reflect the meaning of a sign across all contexts, nor do they give any indication of a token’s grammatical function. For example, the ID gloss england is used for the sign which can mean ‘England’ or ‘English’, regardless of whether the token in question is functioning as a noun or adjective or whether it refers to the country, culture, language, etc. (for more on lemmatisation principles, see Cormier et al., 2012).

Decisions about which 10 sign tokens to include or exclude from each participant were based on a number of factors. Firstly, only signs that were found listed in the Brien (1992) Dictionary of British Sign Language/English were included. Some signs with a 1 handshape were excluded from this study. These included signs that involved a change from the 1 handshape to some other handshape in citation form (e.g., the compound signs believe and check) and classifier signs where the handshape can be regarded as an independent morpheme. Like the ASL study, we also ignored thumb-only variants that functioned as non-first person pronominal pointing signs. In these cases, we assumed that the variation in handshape could also be conditioned by the seating arrangement for filming (participants were seated close together and angled such that they were nearly facing each other. In this situation, pointing to the extreme left or right of the signing space towards the addressee was unlikely). We did, however, include the thumb-only variant in first person pronominal points. In addition, we did not include any signs in which the handshape was obscured. The set-up of the filming studio and the three cameras in use during filming afforded us two views of the signer: one camera had both participants in view and each of the remaining two was directed towards one individual in the pair, so we were able to see the hand configuration used in almost all cases. In order to control for frequency of sign tokens, so that a single sign was not over-represented in our final dataset of tokens and to ensure that we had a good mix of individual items, we employed a ‘three strikes’ rule to token collection. If a single sign occurred three times within one participant’s dataset, then all further instances of that sign were ignored. Coding began several minutes into filming so that participants had time to relax in the presence of the cameras. To summarise, after the first three minutes, the first 10 signs to appear that used the 1 handshape in its citation form and fitted our criteria (i.e., it was in the Brien (1992) BSL dictionary, it was not a compound or a classifier sign, and it had not appeared more than three times) were included in the study.

The procedure described above produced an initial dataset of 2110 tokens. However, some signs were excluded from the final analysis reported here because we were later unable to determine with certainty whether a given sign had the 1 handshape as its citation form or because we were unable to resolve lemmatisation issues (i.e., whether or not a pair or set of sign variants with similar form and similar meaning should be regarded as separate lexemes). Additionally, as the work conducted here complements concurrent work on a new BSL dictionary, ongoing lemmatisation has revealed evidence suggesting that some signs initially coded as having the 1 handshape in citation form in fact did not (see Cormier et al. (2012) for information on the criteria used to decide a sign’s citation form). For example the sign ID glossed as slow can be articulated using the 1 or B handshape in its citation form. For these reasons, a total of 17 sign types representing 26 tokens (approximately 1% of the total dataset) were excluded from the analysis reported below leaving 2084 tokens remaining.

During coding, each token was coded for the following linguistic factors: handshape category, grammatical category, indexicality, lexical frequency, and handshape of the preceding and following sign. Each was also coded for the social factors described above: gender, age, region, social class, ethnicity and language background. A subset of the pointing signs (i.e., the prononimals and the locatives) were coded for orientation of the hand, and for the orientation of the preceding and following sign. For the category of handshape, each token was placed into one of eight categories represented in Table 2. This handshape table attempts to account for all the possible handshape variants that may be observed in signs specified for the 1 handshape. The handshapes shown in Table 2 do not reflect all of the possible variants in the 1 handshape. As will be seen in the results section, however, the majority of handshape variants are restricted to four categories. The division of categories was partly motivated by the presence or absence of four features: open or closed thumb, open or closed unselected fingers, open or closed index finger, and bent or straight index finger.

For the purposes of this study, we judged a sign as being in citation form when the sign was produced with the index finger fully extended, the unselected fingers closed, and the thumb held over or alongside the closed unselected fingers (i.e., the ‘c’ form shown in Table 2). Please note that, unlike the examples provided in Table 2, not all sign variants deviating from citation form were fully articulated. A form of a sign was categorised as the ‘L’ variant, for example, when the thumb was no longer touching the unselected fingers, regardless of whether it was fully extended or not. Additionally, the ‘5’ variant did not require all five digits to be fully extended. The index, middle finger and thumb could be extended and the ring and pinky finger closed and the sign would still be classed as ‘5’. Therefore, sign tokens were grouped into the feature category that best represented the token’s handshape (according to +/− thumb, +/− unselected fingers, etc.), and the forms shown in Table 2 are simply exemplars of each of these categories. Given the large number of handshapes with varying degrees of finger and thumb extension and the complexity of coding required to represent this accurately, we adapted the simplified coding system used by Bayley et al. (2002) in an attempt to simplify annotation of the data.

For grammatical category, signs were coded into the major content grammatical categories of noun (e.g., girl), verb (e.g., think), adjective (e.g., easy), adverb (e.g., tomorrow), depending on their use in sentential context. Function words included pronouns (e.g., pt:pro1), wh-question signs (e.g., why) and other functors (e.g., but). As in a previous study (Schembri et al., 2009), we used a number of semantic and morphosyntactic criteria as the basis for our categorisation of sign tokens into these grammatical categories, but in some cases, it was not easy to determine the grammatical function of a specific token. This was more often the case for pointing signs compared to other sign types. Although first person pronouns were unambiguous (as was often the case for second person pronouns), third person pronouns and locative points (i.e., adverbials) proved difficult to distinguish (there is some discussion in the literature about whether strictly pronominal functions for pointing can be distinguished from other uses of pointing in sign languages or indeed in gesture, see Cormier et al., submitted for publication; Evans and Levinson, 2009; Johnston, in press). Third person pronouns were typically identified as a point to the peripheral signing space, away from the conversational partner, and serving as a referent for another person in the discourse. Locative points were identified as a point to a location (e.g., associated with a place-name mentioned in the discourse) or the location at which a topic being discussed was situated. Ambiguous points were often points to a location associated with a referent in a particular location (e.g., person standing in a doorway), making it difficult to determine whether they were primarily locative or pronominal pointing signs. Consequently, these ambiguous points were ignored. Pointing signs functioning as determiners were identified by their syntactic position adjacent to nominal signs and by prosody. If the point (whether it occurred before or after a noun) could be grouped with a noun as a single cohesive prosodic unit, then it was classed as a determiner.

For indexicality, sign tokens were grouped into the following two categories: pointing signs and non-pointing signs. Pointing signs were further subdivided into pronominals (first, second, and third person pronouns, both singular and plural forms), as well as locative points (e.g., pt:loc meaning ‘there’ or ‘here’), determiners (e.g., pt:det meaning ‘that’ or ‘the’), and other uses of pointing (e.g., pt:other which were often temporal points). Thus, the category of pointing signs includes both signs that function as grammatical signs (e.g., pronouns and determiners) as well as signs that may be considered as content signs (e.g., pt:loc can function as an adverb meaning ‘there’).

Initial results indicated that grammatical category and indexicality interacted to a significant degree (i.e., one of the most frequent grammatical categories in the data, pronouns, consisted exclusively of pointing signs) so it was decided to merge the two factor groups into one.

As indicated in previous studies on spoken languages (e.g., Bybee, 2002; Philips, 1984), lexical frequency may be a factor in phonological variation. That is, high frequency words may exhibit greater phonological variation than low frequency words. As such, we coded as ‘high frequency signs’ in our data all tokens of the top five most frequent items produced with a 1 handshape in a related study of lexical frequency in the BSL Corpus conversational dataset (Cormier et al., 2011): pt:pro1, pt:pro3, pt:pro2, pt:det and pt:loc. Together these 5 lexical items represented 1061 tokens, or 51% of our entire dataset. All other tokens (representing the remaining 87 lexical items) were coded together as ‘not high frequency signs’.

To investigate the effects of the immediate phonological environment, the preceding and following signs were examined and grouped into a category exhibiting the 1 handshape, or exhibiting a different handshape. If no sign immediately followed or preceded the target sign (e.g., when the target sign represented the first or last sign in the participant’s turn in the conversation), then we coded this as a ‘pause’. We only coded the handshape of preceding/following signs using the same hand as the target sign (regardless of whether there was a shift in dominance).

**Fig. 2:**
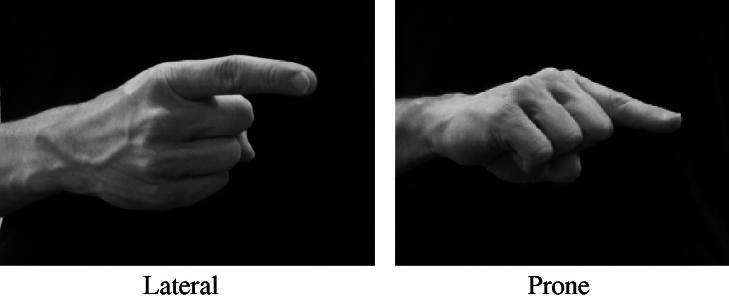
Two categories of orientation used to group pronominal and locative signs.

For the orientation study, all non-first person pronoun signs and locative pointing signs were further categorised according to orientation: as ‘lateral’, ‘prone’ (see Fig. 2 above) or ‘other’. We also coded whether the orientation of the preceding and following signs matched the orientation of the target sign.

The data were coded by the first and third authors, both of whom are deaf native/fluent signers of BSL. Firstly, ten tokens were identified by one researcher and preliminarily coded for all linguistic factors discussed above. The second researcher then examined all the tokens coded in each ELAN file and checked for accuracy and consistency of coding based on the criteria noted above. Any disagreements about coding were discussed, and only tokens in which agreement was reached were included in the analysis. Following completion of coding, 10% (*n* = 210) of the initial data coded were examined by the second author, a hearing fluent user of BSL, to determine inter-rater reliability. All categories within each token were checked (e.g., handshape, grammatical category, orientation, etc.) and the researcher indicated whether he agreed with the coding assigned or not. This produced an agreement level of 96% (i.e., only 4% of categories being ones that he would have coded differently). Social factors were coded by referring to the demographic questionnaire that each participant filled in prior to taking part.

For the statistical analysis, we used the variable rule program Rbrul (Johnson, 2009) which enables us to quantitatively determine the effect of several factors on a binary linguistic variable. In the main analysis, these factors may be linguistic (e.g., grammatical category, the handshape of the preceding sign, etc.) or social (e.g., gender, age) and the binary linguistic variable can be understood as whether the target sign exhibited the 1 handshape or some other handshape (in the other analyses reported here, the linguistic variable might also be a different handshape variant or a specific orientation). Rbrul was designed to improve upon Goldvarb (used extensively in sociolinguistic research, see Tagliamonte, 2006) in that it not only considers fixed effects (such as the linguistic and social factors mentioned above) but random effects as well. In this study, two possible random effects are participant and lexical item. Strictly speaking, tokens are not independent of one other and may be grouped according to these random effects (e.g., 10 tokens can have the same participant in common and, as we see below, 370 tokens have the same sign in common). These random effects themselves may favour or disfavour a variant beyond what can be attributed to fixed effects alone (e.g., a specific lexical item that strongly disfavours handshape variation may appear as a gender effect if the same item is particularly common amongst women in our data). In considering this, Rbrul reports significant results on a given variable only when they are strong enough to discount any possible influence from random effects (something that GoldVarb does not do). A further advantage of using Rbrul is that results are presented in both factor weights, which are used frequently by sociolinguists when displaying results from studies using variable rule programs, and log-odds, which have the benefit of being understood by the wider research community (such as psycholinguistics, psychologists and statisticians).

## 5 Results

### 5.1 Distributional results

Table 3 provides an overview of the type and frequency of tokens collected for the study together with the number of times each type appeared in citation form.

Table 3 shows that our collection of 2084 sign tokens contains 92 sign types (i.e., lexical items), each having the 1 handshape in its citation form. Additionally, the figures in Table 3 indicate that the top ten most frequent signs in our data account for 72% of the total number of tokens (*n* = 1503). When our calculations are expanded to include the top 25 most frequent signs in our data, this figure increases to 89% (*n* = 1850). Finally, Table 3 indicates that 97% of the 2084 sign tokens (*n* = 2016) is represented by 50 unique sign types.

Although corresponding lexical frequency information is not available for ASL, Bayley et al.’s (2002) collection of sign tokens have been grouped by grammatical category. In Table 4, we have compared the distribution of grammatical category in the current study with ASL. We can see, therefore, that the most frequent lexical item pt:pro1 represents a considerably smaller percentage of the BSL dataset (18%) than the ASL dataset (39%).^4^ Indeed, Table 4 shows that the proportion of all pronominal signs is also smaller overall in BSL (42%) than in ASL (57%). This is likely the result of our upper limit on the number of tokens of the same sign that could be coded in each participant’s data. Overall, the distribution of sign tokens by grammatical category is more even in the current study compared to the ASL study which shows a strong bias towards first person pronominals.

It is worth noting that pointing signs in general account for the majority of the overall data. Pointing signs include not only pronominal signs but also determiners (pt:det) and adverbials (pt:loc ‘there’). When a distinction between pointing and non-pointing signs is applied to the BSL data, we find that pointing signs represent 55% (*n* = 1146) of 2084 tokens.

### 5.2 Handshape variation

A phonological analysis of 2084 sign tokens reveals that 53% (*n* = 1127) used a handshape that deviated from citation form. In Table 5 below, the distribution of tokens according to the handshape variant used is provided.

Table 5 shows that 98% of the BSL data can be identified as belonging to four handshape categories: citation form (46%), the 5 variant (20%), the L variant (16%), and the 4 variant (15%). In the ASL study, 96% of their data consisted of all these variants although the 4 handshape variant was not a major category in their study (accounting for only 4% of 5356 tokens). The proportion of tokens identified as citation form in the ASL study is slightly less than the BSL dataset (39%) whilst the proportions of the L variant and 5 variant are slightly higher in the ASL dataset (29% and 25%, respectively). Results from both datasets strongly suggest that variation in the 1 handshape is a highly frequent phenomenon accounting for the majority of tokens.

The results of our statistical analyses are presented below.

### 5.3 Statistical analysis of linguistic factors

All the linguistic factors we analysed were significant in the following order of importance: (1) preceding handshape or pause, (2) following handshape or pause, (3) grammatical category and indexicality, and (4) lexical frequency, as shown in Table 6. For ease of analysis, since Rbrul requires a binary or a continuous application value, the categories of handshape were divided into two: citation form and other handshape. The application value selected in all cases below was ‘other handshape’. Therefore, log odds and factor weights should be understood as reflecting the likelihood that a given factor might influence the appearance of non-citation forms.

Table 6 demonstrates that the immediate phonological environment plays a significant role in the variation observed within our data. In other words, statistical analysis revealed the preceding phonological environment to be the most important factor influencing handshape variation in signs with the 1 handshape in citation form. If the preceding sign featured a handshape that was not the 1 handshape (0.747) or if a pause preceded the target sign (0.345), then it was very likely that we would also observe some other handshape in the target sign. Conversely, if the preceding sign included a 1 handshape, then it was very unlikely that we would see a different handshape in the target sign (−1.092). Similar results were found for the following phonological environment which was the second most important factor. That is, if the following sign featured a handshape that was not the 1 handshape (0.374) or if a pause followed the target sign (0.417), then it was very likely that the target sign would anticipate features in the following sign. As with the preceding sign factor group, if the following sign had a 1 handshape, it was very likely no variation in the target sign would be observed (−0.791). The next most important factor was the combined factor groups of grammatical category and indexicality. Within this factor group, singular pronouns are most likely to exhibit variation over all other categories, with pt:pro1 the most variable (2.381), followed by pt:pro2 (0.760) and then pt:pro3 (0.726). This group of pointing signs, along with pointing signs that act as determiners (0.613), all favour handshapes other than the 1 hand configuration. Not all pointing signs display this tendency; pointing signs glossed as pt:other slightly disfavour variation (−0.096) as do locative points (−0.211) and plural pronouns (−0.404). However, within the non-pointing signs, we see an overall tendency to disfavour variation beginning, very weakly, with wh-signs (−0.012) and followed by adverbs (−0.390), adjectives (−0.576), other functors (−0.659), verbs (−1.023) and nouns (−1.109). Finally, lexical frequency also returned a significant result, although this result was obtained in a separate Rbrul analysis that excluded grammatical cateogry and indexicality, as once again, initial analyses indicated that these two factor groups interacted to a significant degree. The top 5 most frequent signs (based on a separate lexical frequency study; see Cormier et al., 2011) that have the 1 handshape as citation form were significantly more likely to display 1 handshape variation (0.791) when compared to less frequent signs (−0.791).

The interaction between the three original factor groups (grammatical category, indexicality and lexical frequency) is an important finding in itself, as previous work on ASL (e.g., Bayley et al., 2002) did not fully incorporate either indexicality or lexical frequency effects into their analysis. The interaction reflects that fact that pronouns (the grammatical category that most strongly favours handshape variation) are pointing signs that are, additionally, highly frequent. This underlying relationship between factor groups had a significant effect on the statistical analysis as Rbrul assumes factor groups to be independent from one another, and this is what motivated our merging two groups (grammatical category and indexicality) and carrying out a separate analysis with another (lexical frequency). We wished to demonstrate that there are multiple linguistic effects that appear to be driving the variation in the BSL data.

In Table 7, we report a detailed analysis of the preceding and following phonological environments conditioning the use of the citation form of the 1 handshape. Preceding and following pauses both somewhat disfavour the use of a citation form (in the log odds column, a result below 0 shows a tendency to disfavor the 1 handshape whilst a result above 0 shows a tendency to favour the 1 handshape).

The results in Table 7 demonstrate that, for the preceding handshape, the occurrence of another citation handshape (1.197), or the related variant X (1.029) in which the index finger is extended and bent, both strongly favour the use of a citation form. The other handshape category also favours the use of a 1 handshape (0.280). Preceding handshapes which disfavour the citation form involve the extension of the thumb, such as 6 (−0.107), & (−0.438) and L (−0.773), and those which involve the extension of the middle, ring or pinky fingers, such as 5 (−0.518) and 4 (−0.432). A preceding pause also disfavours the citation form (−0.239). For the following handshape, the conditioning factors are different, with only the 1 handshape (0.974) strongly favouring the use of citation form. The & variant (0.030) slightly favours the citation form, while, rather unexpectedly, all other following handshape variants disfavour the use of the 1 handshape.

### 5.4 Social factors

In contrast to linguistic factors, only one social factor returned a significant result in the main analysis: region. Gender, ethnicity, age, language background and social class were not significant factors affecting 1 handshape variation. Table 8 displays the results from the main Rbrul analysis for social factors. As with the previous table, the application value selected is ‘other handshape’. Therefore, the log odds and factor weights reflect the likelihood that some other handshape would be observed in place of the 1 handshape.

Table 8 shows that, out of the seven regions included in the analysis, three regions appear to favour handshape variation. Specifically, participants from Cardiff were most likely to exhibit handshape variation (0.516). Participants from Belfast (0.211) and Bristol (0.205) may be said to somewhat favour handshape variation. Manchester participants were least likely to display variation in the 1 handshape (−0.443) followed by Birmingham participants (−0.324). Glasgow participants (−0.142) and London participants (−0.022) appear to slightly disfavour variation in handshape.

### 5.5 Additional analyses involving other handshape variants as the dependent variable

Given the relationship between the L and 5 variants and specific social factors reported in the ASL study, we also ran analyses in which the two main subvariant handshapes (i.e., 5 and L) were the dependent variable. The significant factor groups are reported in Tables 9 and 10 respectively.

As with the main analysis (where the 1 handshape was selected as the dependent variable), the preceding and following phonological environments are the most important factors in conditioning the use of a 5 handshape variant in the target sign, with a preceding (1.047) or following (0.829) 5 variant being the most important factor. Preceding (0.603) and following (0.573) pauses also favour variation. In addition, we can see that handshapes in the preceding sign that involve thumb extension, such as L (0.166) and 6 (0.039), tend to favour the use of a 5 variant. In contrast, the & variant, which also involves thumb extension, appears to disfavour the 5 variant (−0.543). The 4 variant, typically involving extension of the middle, ring or pinky fingers, appears to weakly disfavour the 5 variant (-0.014). All variants with no thumb and finger extension, such as 1 (−1.202), and those with bending of fingers at the joints, such as X (−0.156) tend to disfavour the 5 variant. Similar results can be seen in results for the handshape in the following sign. All variants with no thumb and finger extension, such as 1 (−1.032) and X (−0.070) appear to disfavour the 5 variant whilst the results for the other variants appear to be mixed (e.g., the appearance of the L variant (−1.073) in the following sign strongly disfavours the 5 variant whilst other variants involving thumb extension such as the & (0.295) and 6 (0.118) variant display the reverse). Grammatical function and indexicality is also a significant factor group conditioning variation, with the pronominal signs, such as pt:pro1 (2.036) and pt:pro2 (0.816), and wh-signs (0.083) all favouring the use of the 5 variant, and other sign types disfavouring it. We also see that region is significant, although differing slightly in detail from the results in the main study: signers in Cardiff (0.440), Bristol (0.378), Belfast (0.216), London (0.102) and Glasgow (0.034) tend to favour the 5 handshape variant, while those in Manchester (−0.442) and Birmingham (−0.728) disfavour it.

For the L variant (see Table 10), again we see that the preceding and following phonological environments are the most important factor groups in predicting the presence of this variant (with stronger effects than for the 5 handshape), with a preceding (2.182) or following (0.923) L strongly favouring the L variant in the target sign. Moreover, we see that handshapes in the preceding sign that involve thumb extension, such as & (2.388) and 6 (2.187), and those variants with extension of the index finger, such as 5 (1.338), 1 (0.832) and 4 (0.601), tend to favour the use of a L handshape. This effect is not found in the following phonological environment, where only the presence of a 6 handshape (0.767) favours this variant. Preceding (1.323) and following (0.123) pauses also favour an L variant. In contrast, a preceding X handshape, and almost all following handshapes, disfavour the L variant. Finally, none of the social factors reaches significance in this analysis.

These results (combined with the results reported in Section 5.4) are interesting when we consider the complex picture reported in the ASL study regarding social factors and 1 handshape variation. Across all analyses, only one social factor appears to be important in the BSL dataset: region. These differences and the possible reasons behind them are discussed in Section 6.

### 5.6 Orientation of pointing signs

An analysis of 646 pointing signs for orientation (focusing on non-first person pronominal and locative pointing signs) suggests systematic variation in orientation according to a pointing sign’s underlying function. As we made a tripartite distinction in the orientation of pointing signs (lateral, prone and other), two statistical analyses are reported below (as Rbrul was used to run analyses with a binary application value). These are: lateral versus all other orientations and prone versus all other orientations. The results of the analysis are reported in Table 11 and 12. In these tables, the application value is ‘vertical orientation’ and ‘horizontal orientation’ respectively.

For both analyses, only one linguistic factor was found to be significant: lexical item. The orientation of the preceding and following signs were not found to be significant. In Table 11 (lateral orientation versus other orientation), the results demonstrate that pt:pro2 (0.782) and pt:pro3 (0.656) are likely to use a lateral palm orientation whilst pt:loc (−0.555) and pt:pro2/3pl (−0.719) display the reverse (i.e., are likely to use a palm orientation that is not lateral). Table 12 demonstrates that the reserve is true when we group tokens into those that exhibit a prone orientation and those with any other orientation. That is, pt:pro2/3pl (0.931) and pt:loc (0.581) are likely to use a prone palm orientation whilst pt:pro2 (−0.626) and pt:pro3 (−0.730) display the reverse. In both orientation analyses, no social factors were found to be significant.

## 6 Discussion

Our results suggest that, like ASL, variation in the 1 handshape is not random but can be linked to linguistic and social factors. Also, factors such as grammatical category and features of the surrounding phonological environment were found to characterize variation in the 1 handshape in BSL, similar to ASL (Bayley et al., 2002). However, our results revealed some interesting differences compared to Bayley et al. (2002). Firstly, indexicality and lexical frequency are important factors in the BSL data; these factors were not considered in the ASL study. Secondly, only one social factors returned a significant result (region), compared to multiple social factors that had a significant effect in 1 handshape variation in the ASL study. Finally, the orientation analysis for BSL suggests that singular pronominal pointing signs strongly favour lateral orientation and locative signs strongly favour prone orientation. These results, whilst providing additional support to claims made within the existing sign language literature, bring to light several questions that will be discussed in detail below.

### 6.1 Linguistic factors

Our results indicate that all the linguistic factors considered here have a significant influence on variation in the 1 handshape. These factors, listed in the order of importance, are the preceding handshape, the following handshape, grammatical category and indexicality, and lexical frequency. These linguistics factors are discussed in turn below.

#### 6.1.1 Preceding and following phonological environment

In Bayley et al. (2002), features of the preceding and following phonological environment as well as grammatical category were found to be important factors although grammatical category was the first-order constraint governing variation in the 1 handshape in ASL. However, in our study, the preceding phonological environment was the first order constraint on variation in the 1 handshape followed by the following phonological environment. Instead, the combination of grammatical category and indexicality was the third most important constraint on variation in the 1 handshape. The categories of the preceding and following phonological environment each consisted of three subcategories: signs that also had the 1 handshape, signs with some other handshape, and pauses. Our analysis revealed that preceding or following signs with some other handshape as well as pauses pattern similarly (and in contrast to preceding or following signs with the 1 handshape) in that they tend to favour variation in the target sign. We also found features of the preceding and following phonological environment to be important when either the 5 or L variant was selected as the dependent variable and it was the only linguistic factor that was significant across all the analyses. A detailed analysis examining each handshape variant revealed, as in the ASL study, that features of the preceding and following handshape generally favour similar features in the target sign. For example, the presence of a 1 handshape variant or the related X variant (with an extended and bent index finger) in the preceding sign both strongly favour the appearance of a 1 handshape variant in the target sign. This is further supported by the results presented in Table 10 where the L variant is selected as the dependent variable. Here, all handshape variants which do not have thumb extension (as is present in the L variant) in the preceding sign disfavour the appearance of the L handshape in the target sign. In other words, and as has been reported for ASL in Bayley et al. (2002), the absence of an extended thumb in the preceding handshape favours the absence of an extended thumb in the target sign. However, we do observe mixed results that point away from a systematic relationship between features in the surrounding phonological environment and features in the target sign. For example, in Table 9, where the 5 variant has been selected as the dependent variable, the 4 handshape variant in the preceding sign slightly disfavours the appearance of the 5 handshape in the target sign and other handshapes that have fewer features in common with the 5 variant (such as the 6 or L variant) are reported to have the opposite effect. These results may be linked to the research methodology involved in grouping tokens into their respective handshape categories. For example, a given token does not need to have all four digits fully extended to be labelled as the 4 variant. If the pinky finger along with the index finger were fully extended, this would be enough to label the token in question as the 4 variant. For this variant, the degree in which the pinky finger is open was observed to vary greatly and therefore should not be regarded as fully extended in each instance. In fact, it should not generally be assumed that full extension of the relevant digits was observed in each token within their respective categories. A more detailed phonetic analysis is required to fully appreciate what may be happening here. However, our results generally support that which is reported in Bayley et al. (2002): features of the surrounding phonological environment tend to favour like features in the target sign.

#### 6.1.2 Indexicality

Bayley et al. (2002:46) observed that there appeared to be a relationship between indexicality and grammatical function in their data, noting an apparent relationship between handshape variation in pronominal signs and a “…continuum of distance from the signing subject in the discourse setting”. They found that most variation in handshape occurs in the first person pronoun, which we also find here. Bayley et al. suggest that this is because the most salient referent in the discourse is the signer, and thus this referent is most readily identified by a range of handshapes directed towards the signer’s chest. Bayley et al. also observe somewhat less handshape variation in second person pronouns (typically a point towards the addressee) which they describe as a point to the second most salient referent. Thirdly, Bayley et al. note that non-addressed referent(s) are the third most salient referent(s), and relatively less variation may be expected, as does indeed appear to be the case for ASL. The results in the current study do indicate a similar pattern regarding indexicality in BSL. That is, we do observe a systematic pattern where sign types that may be regarded as more indexic than others also exhibit a higher degree of 1 handshape variation over less indexic sign types. As in the ASL study, we found the most variation in the first person pronoun, the sign type regarded as most indexic. The second person pronoun is the second category in which we observe the most variation, and a category that might be considered less indexic than the first person pronoun, followed by the third person pronoun. It must be said, however, that the statistical analysis reveals that these two categories display a very similar level of variation with the third person pronoun showing only slightly less variation (0.726) than the second person pronoun (0.760). Additionally, we see more variation amongst wh-signs (a category of non-indexic signs) than in the category of locative pointing signs, for example, which suggests that the relationship between indexicality and variation may not be as straightforward as previously assumed. However, plural pronouns forms, which have been independently argued to be less indexic than other pronouns (Cormier, 2002, 2007), disfavour variation contrasting with singular (and more indexic) pronouns which do favour variation. Bayley et al. (2002:46) propose that, in pointing signs, “…the indexical function is carried by the tips of the fingers, regardless of the handshape used. In nonindexical lexical signs, however, the whole handshape carries part of the semantic load. The handshape in this class is the most likely to be the citation form.” The BSL data appears partly to support this claim, in which most pointing signs favour variation compared to most non-pointing lexical items favouring citation forms.

#### 6.1.3 Grammatical category

The influence of grammatical category on 1 handshape variation was considered together with indexicality for each of the statistical analyses performed here. Within these analyses, it is possible to see a division between content and functional signs. In the main statistical analysis, functional signs such as the first, second and third person singular pronouns and determiners all strongly favour handshape variation. In contrast, all signs explicitly identified as content signs (i.e., nouns, verbs, adjectives, adverbs) consistently favoured use of the 1 handshape. The fact that locative points also appear to favour the 1 handshape reflects the fact that locative points may have an adverbial function (e.g., meaning ‘there’ or ‘here’). However, wh-signs, plural pronouns and other functors (e.g., but) appear not to follow this pattern with wh-signs weakly favouring the 1 handshape, followed by plural pronominals and other functors (in fact, other functors favoured the 1 handshape more strongly than adverbs and adjectives).

It is important to consider the finding that singular pronouns and determiners strongly favour variation together with the fact that the immediate phonological environment plays an important role in conditioning this variation. These combined factors lend support to observations within a number of sign languages that the handshape of pronouns, which are typically unstressed, frequently assimilates to that of a neighbouring lexical sign (see Sandler and Lillo-Martin, 2006) which has been argued to be due to cliticisation (Sandler, 1999). Cliticisation is also a likely explanation for assimilation patterns with determiners which are in a syntactically weak position. This may explain why locative points, if they differed in their syntactic distribution, patterned differently to pronouns and determiners in the main analysis. Although further research is required, one might also speculate that our results reflect language change in progress where pronouns or determiners are forming constructions with highly frequent nouns, verbs or other lexical items in grammaticalisation processes, much in the same way as has been reported for spoken languages such as English (e.g., how frequent constructions such as ‘*let us*’ has led to the first person plural pronoun being cliticised in ‘*let’s*’ and that this construction has led to the formation of a single word ‘*lets*’) (Hopper and Traugott, 2003).

#### 6.1.4 The sign language lexicon and gesture

The findings from this study have implications for our understanding of the sign language lexicon, in particular the role of gesture within the lexicon. Following Brentari and Padden (2001), signs belonging to the native component of the lexicon can be divided into core (or frozen) lexicalised signs (i.e., signs that have a consistent form and meaning relationship and abide by a set of phonological constraints) and non-core signs (i.e., signs that have their roots in gesture and are only partly specified in one or more but not all of the phonological parameters). Pointing signs represent an important part of the non-core native lexicon since they are only lexically specified for handshape. Given that the 2084 tokens analysed in this study can be clearly divided into core and non-core signs with the latter consisting solely of pointing signs, one can see that there is an overall tendency for signs from the non-core component of the sign language lexicon to strongly favour variation. Therefore, the fact that pointing signs pattern differently to signs belonging to other components of the sign language lexicon (e.g., the core lexicon) suggests that they have different underlying phonological properties to these signs (as proposed in Brentari and Padden, 2001). On closer inspection, however, locative points and plural pronominals appear not to follow this pattern since both categories of pointing signs differ from other pointing signs by disfavouring handshape variation. This inconsistency can be partly reconciled with the division suggested here by arguing that plural pronominals are more lexicalised than other types of pointing (see Section 6.4) and therefore may be expected to behave similarly to core lexical signs. However, this explanation cannot be equally extended to locative points.

One way to further understand the nature of locative points and their position within the sign language lexicon (particularly in relation to other uses of pointing) is by comparing them with locative pointing in gesture by non-signers. In Section 2.4, we demonstrated this approach by comparing the findings of Cooperrider (2011) and Bayley et al. (2002) with respect to first person pronouns and found that a higher rate of variation can be seen in gestural ‘self-points’ (with or without thumb extension) (90%) when compared to first person pronouns in ASL (52%). Interestingly, the rate of variation in first person pronouns in BSL (60%) is similar to that observed in ASL and lends further support to the claim that first person points in signed languages are more lexicalised (at least for handshape) when compared to self-points in gesture (Meier, 1990; Meier and Lillo-Martin, 2010). Returning to locative signs, we do not have similar comparative data available to draw a comparison with locative points in gesture (nor do we have comparative data for other pointing types). Whilst descriptive accounts have demonstrated similar patterns in orientation in locative versus personal points in spoken co-speech gesture (Kendon and Versante, 2003), further research is needed that quantitatively outlines handshape and orientation variation in gesture by non-signers. Findings from these studies will enable us to understand why locative points pattern differently from pronominals and where they exist within the sign language lexicon. It may be that, as noted above in Section 6.1.3, locative signs simply block handshape/orientation assimilation effects (due to appearing in stressed positions unlike singular pronominals and determiners) but that this does not affect their position in the non-core lexicon.

#### 6.1.5 Lexical frequency

Using data from a separate lexical frequency study based on BSL (Cormier et al., 2011), the top 5 most frequent signs that have the 1 handshape as its citation form were identified. The signs identified were pt:pro1, pt:pro3, pt:pro2, pt:det (a pointing sign that functioned as a determiner), and pt:loc (a locative pointing sign). These signs represented 51% of our dataset. When we compared the likelihood that these signs would exhibit handshape variation over all the other 87 signs in our data, we found that handshape variation was significantly more likely in these 5 signs. This finding indicates that frequency is an important factor to consider when investigating phonological variation. This is supported by similar findings in another study looking at phonological variation in the location parameter in Auslan and NZSL (Schembri et al., 2009). This study found that lowering (in signs produced at the forehead in citation form) was significantly more likely to be seen in highly frequent verbs than other sign types. Despite a dearth of sign language studies that consider the effect of frequency on phonological variation, the location variation study reported by Schembri et al. (2009) on Auslan and NZSL and the results from the current 1 handshape study on BSL provide important evidence that underscore its importance, in line with similar claims made for spoken languages (e.g., Guy, 2007). Frequency was not considered as a factor in the ASL study, however, and it is likely that similar frequency effects like those we have observed here may be at work. The three signs that form the majority of the ASL dataset (i.e., pt:pro1, pt:pro2, and pt:pro3 which account for 57% of their data) were also identified as the most frequent signs (pt:pro2 and pt:pro3 were collapsed into a single category representing non-first pronominals) in a small-scale lexical frequency study based on 4111 signs from ASL (Morford and MacFarlane, 2003).^5^

### 6.2 Social factors

Bayley et al. (2002) reported that a number of social factors could be linked to variation in the 1 handshape in ASL. These factors include region, age, language background, social class, and ethnicity (gender was not found to be significant). Their results can be viewed according to two regional groupings with signers from California, Kansas/Missouri, Louisiana and Massachusetts all using significantly more citation forms and signers from Maryland, Virginia and Washington using significantly fewer citation forms. Within the two groups, it was suggested that handshape variation could be linked to the remaining social factors. For example, younger signers from California, Kansas/Missouri, Louisiana and Massachusetts used significantly more citation forms whilst older signers did not. In contrast, this complex patterning regarding social factors and 1 handshape variation was not observed in the BSL study reported here. We found that region was significant in the main analysis. Signers from Cardiff, Belfast and Bristol were more likely to use non-citation forms than signers in Manchester, Birmingham, Glasgow, and London.^6^ Region was also found to be significant in the analysis of variation linked to the 5 variant. Signers from Cardiff, Bristol, Belfast, London, and Glasgow were more likely to use the 5 variant with signers from Manchester and Birmingham more likely to use some other handshape. Age, gender, ethnicity, language background and social class were not significant factors in any of the analyses reported here. Previous studies on sociolinguistic variation in BSL have highlighted regional variation at the lexical level alone (e.g., Stamp et al., in press; Sutton-Spence et al., 1990) so this is the first study to suggest that these social factors may also be relevant for variation at the phonological level in BSL. The dissimilarity between the ASL and BSL studies may be attributed to differences in research design. For the BSL study, we adopted a ‘three strikes’ approach to coding. That is, if we saw the same three signs within the 10 tokens allocated to each participant, we ignored all subsequent tokens of the same sign and sought other signs using the 1 handshape instead. In doing so, we were able to ensure that the 2084 tokens collected were not biased towards a particular sign type. For example, pt:pro1 represents 39% of the ASL dataset and only 18% of the BSL dataset. Additionally, this also ensured that tokens were more evenly distributed across grammatical categories than the ASL study (although our approach still shows a strong bias towards pointing signs in general). It is not clear from the ASL study how likely it is that an individual lexical item (in this case pt:pro1) might have had an effect on handshape variation in the ASL study: what might appear to be a complex picture regarding social factors may, in fact, have an underlying cause in individual lexical items. These effects might then disappear (or be reduced considerably) should one take into account lexical item as a random effect in a mixed effects model, as we have done here. In theory, one could argue that this point extends to the linguistic factors also reported as significant in the ASL study. However, the fact that we find similar linguistic factors to be significant (e.g., grammatical category and features of the immediate phonological environment) lends some support to the idea that these linguistic factors may have an effect on 1 handshape variation across unrelated sign languages.

### 6.3 Orientation

The results from this study provide empirical support to earlier claims made about the systematic variation in the orientation of non-first pronominal and locative points. That is, researchers have claimed that pronominal points are typically articulated with a vertical (or lateral) orientation and locative points are articulated with a horizontal (or prone) orientation. These claims have been made for Kata Kolok (de Vos, 2008), Danish Sign Language (Engberg-Pederson, 2003), and Sign Language of the Netherlands (Nederlandse Gebarentaal, NGT) (van der Kooij et al., 2006), although the NGT study reports that a horizontal orientation is used to localise referents while a variable palm orientation is reported for pointing signs that refer to previously established referents. The findings from the current study indicate that pronominal and locative points pointing signs in BSL do display systematic variation in orientation with non-first pronominal pointing signs favouring a vertical (or lateral) orientation and locative points favouring a horizontal (or prone orientation). We also found that non-first plural points differ from non-first singular points with the former favouring a horizontal orientation. We considered whether other linguistic factors (e.g., the orientation of the preceding and following sign) and social factors (e.g., age, ethnicity, language background, and region) might influence the variation observed in non-first pronominal and locative points but no significant effects were found. That is, it appears from our analysis that variation in the orientation of non-first pronominal and locative points are conditioned by the function of the pointing sign alone. Whilst our findings lend strong support to previous claims in the sign language literature, it is worth remembering that these results show only an overall tendency for non-first pronominal and locative points to favour a specific orientation. In other words, whilst we may expect non-first pronominals to exhibit a vertical orientation and locatives to exhibit a horizontal orientation, not all of them do so. Further analysis of these pointing signs, perhaps to investigate further the claims made by van der Kooij et al. (2006) that variation in orientation may be linked to the introduction of a new location to the discourse, together with comparisons with pointing gestures used by non-signers, will shed further light on palm orientation in pointing signs.

### 6.4 Singular versus plural pronominal signs

The current study has shown that overall pronominal signs disfavour citation form compared to lexical signs which favour the 1 handshape in citation form. Additionally, pronominal signs overall favour lateral palm orientation compared to locative pointing signs. However, these patterns only apply to singular pronominal signs. Plural pronominal signs patterned more like lexical signs (in terms of handshape) and locative points (in terms of orientation) and not like singular pronominal signs. These differences between singular and plural pronominal signs are consistent with previous research showing that plural pronouns (in ASL and BSL) are less indexic than singular pronouns – that is, plurals point towards the location(s) associated with their referents less than singulars do (Cormier, 2002, 2007). The fact that plural pronominal signs in this study differed significantly from singulars both in terms of handshape and orientation suggests that it may be better not to consider plural pronominal signs to be pointing signs, at least not to the same extent that singular pronominal signs are, although comparative work on pointing gestures used by non-signers is important here.

## 7 Conclusion

The results of this study indicate that, as in ASL, variation in the 1 handshape is systematically constrained by a range of linguistic and social factors in BSL. Unlike in ASL, however, we find that, apart from the effects of lexical items and individual signer, the surrounding phonological environment is the most significant factor predicting whether a 1 handshape sign will appear in citation form or not, although grammatical function and indexicality are also significant factors conditioning this variation. We find relatively less influence from social factors than was reported for ASL, with only regional differences turning out to be significant overall. These differing results between ASL and BSL may reflect differences in the social structure, history and language attitudes in the different deaf communities, and/or they may partly be the result of different methodological approaches to the study of phonological variation in handshape. Lastly, our findings suggest a role for lexical frequency working in tandem with grammatical function and also with indexicality. It is clear that future studies on phonological variation in sign languages need to take all three of these factors into account.

## Figures and Tables

**Table 1:** Distribution of participants according to six social categories.

Site	Gender		Age		Language background		Social class		Ethnicity	
	F	M	16–50	51+	Deaf	Hearing	Working	Middle	White	Other
Belfast (*n* = 30)	17	13	14	16	7	23	26	4	30	-
Birmingham (*n* = 30)	13	17	18	12	12	18	16	14	27	3
Bristol (*n* = 32)	17	15	15	17	17	15	16	16	30	2
Cardiff (*n* = 29)	17	12	14	15	5	24	21	8	27	2
Glasgow (*n* = 30)	15	15	16	14	6	24	17	13	27	3
London (*n* = 30)	15	15	16	14	12	18	11	19	25	5
Manchester (*n* = 30)	16	14	14	16	9	21	23	7	27	3

Total	109	102	107	104	68	143	130	81	193	18

**Table 2:** Handshape categories representing possible phonological variants of the 1 handshape.^a^

a The handshape images shown in this paper are from the Hamburg Notation System (Prillwitz et al., 1989).

**Table 3:** Distribution of sign types with 1 handshape in citation form and % of citation forms.

Ranking	ID gloss	Number of tokens	% of tokens	Number of + cf	% of + cf
1	pt:pro1	370	17.5	46	12.4
2	pt:pro3	253	12.0	92	36.4
3	pt:pro2	184	8.7	67	36.4
4	pt:loc	155	7.3	92	59.4
5	same	128	6.1	83	64.8
6	pt:det	99	4.7	37	37.4
7	what	98	4.6	37	37.8
8	why	88	4.2	25	28.4
9	one	69	3.3	57	82.6
10	think	59	2.8	38	64.4
11	pt:pro3pl	51	2.4	30	58.8
12	say	45	2.1	27	60.0
13	but	31	1.5	17	54.8
14	time	27	1.3	17	63.0
15	different	25	1.2	12	48.0
16	hearing	23	1.1	14	60.9
17	first	20	0.9	17	85.0
18	england	18	0.9	14	77.8
19	girl	18	0.9	10	55.6
20	go-point	18	0.9	14	77.8
21	pt:other	18	0.9	11	61.1
22	can-not	15	0.7	8	53.3
23	who	15	0.7	13	86.7
24	meet	12	0.6	11	91.7
25	easy	11	0.5	7	63.6
26	pt:pro1pl	11	0.5	6	54.5
27	next-year	10	0.5	8	80.0
28	plus	10	0.5	8	80.0
29	self	10	0.5	9	90.0
30	later	9	0.4	3	33.3
31	thing	9	0.4	8	88.9
32	boss	8	0.4	5	62.5
33	only	8	0.4	4	50.0
34	room	8	0.4	4	50.0
35	talk	7	0.3	6	85.7
36	try	7	0.3	5	71.4
37	most	6	0.3	2	33.3
38	people	6	0.3	3	50.0
39	quick	6	0.3	5	83.3
40	yesterday	6	0.3	5	83.3
41	have-a-look	5	0.2	3	60.0
42	impossible	5	0.2	2	40.0
43	kick	5	0.2	4	80.0
44	teach2	5	0.2	3	60.0
45	university	5	0.2	5	100.0
46	and	4	0.2	2	50.0
47	half2	4	0.2	3	75.0
48	independent	4	0.2	4	100.0
49	other	4	0.2	3	75.0
50	pound	4	0.2	4	100.0
51	bump-into	3	0.1	3	100.0
52	competition	3	0.1	3	100.0
53	information	3	0.1	2	66.7
54	last-year	3	0.1	2	66.7
55	late	3	0.1	1	33.3
56	mention	3	0.1	2	66.7
57	near	3	0.1	3	100.0
58	tomorrow	3	0.1	1	33.3
59	up	3	0.1	1	33.3
60	each	2	0.1	2	100.0
61	germany	2	0.1	1	50.0
62	go-to-and-fro	2	0.1	1	50.0
63	london	2	0.1	2	100.0
64	meeting	2	0.1	2	100.0
65	multiply	2	0.1	2	100.0
66	pt:locpl	2	0.1	0	0.0
67	pt:pro2pl	2	0.1	1	50.0
68	allow	1	0.0	1	100.0
69	always2	1	0.0	0	0.0
70	boy	1	0.0	1	100.0
71	cry	1	0.0	1	100.0
72	cute	1	0.0	1	100.0
73	debate	1	0.0	1	100.0
74	decide	1	0.0	1	100.0
75	escape	1	0.0	1	100.0
76	follow	1	0.0	1	100.0
77	follow2	1	0.0	1	100.0
78	happen	1	0.0	1	100.0
79	knit	1	0.0	1	100.0
80	last-week	1	0.0	1	100.0
81	mouse	1	0.0	1	100.0
82	opposite	1	0.0	0	0.0
83	profile	1	0.0	1	100.0
84	pt:detpl	1	0.0	0	0.0
85	rule	1	0.0	1	100.0
86	russia	1	0.0	0	0.0
87	sack	1	0.0	0	0.0
88	sight	1	0.0	1	100.0
89	summon	1	0.0	1	100.0
90	town2	1	0.0	1	100.0
91	warn	1	0.0	1	100.0
92	week	1	0.0	1	100.0
		2084		959	

**Table 4:** Distribution of sign tokens by grammatical category in BSL and ASL (Bayley et al., 2002).

Grammatical category	BSL	BSL (%)	ASL	ASL (%)
Noun, adjective	301	14.4	419	8.1
Verb, adverb	344	16.5	1238	23.8
Grammatical function sign	390	18.7	501	9.6
Wh-sign	178	8.5	101	1.9
pt:pro3	304	14.6	435	8.4
pt:pro2	186	8.9	482	9.3
pt:pro1	381	18.3	2019	38.9

Total	2084	100.0	5195	100

**Table 5:** Distribution of tokens by handshape displayed in BSL and ASL (Bayley et al., 2002).^a^

Handshape category	BSL (total)	BSL (%)	ASL (total)	ASL (%)
c	959	46.0	2067	38.6
X	22	1.1	60	1.1
L	342	16.4	1556	29.1
4	311	14.9	193	3.6
5	425	20.4	1319	24.6
thumb-only variant (6 and & combined)	21	1.0	161	3.0
o	4	0.2	(did not include as a category)	(did not include as a category)

Overall total	2084	100	5356	100

a Handshape categories used in the study reported here differ slightly from Bayley et al. (2002). The following categories are identical across the two studies: citation form, ‘L’, ‘4’, ‘5’, ‘X’. The open-thumb category in the ASL study is equivalent to the categories of ‘6’ and ‘&’ combined in the current study (the open-thumb category was excluded from the final analysis in the ASL study because it was constrained by the physical location of the referent in question when acting as a pronominal point whereas we ignored similar tokens during the coding process). Finally, we included an additional category ‘o’ for variants that could not be placed into any of the categories previously mentioned. No such category is reported in the ASL study.

**Table 6:** Linguistic factors conditioning variation in the 1 handshape.

Factor group	Factor	Log odds	Tokens	Percentage of application value [other handshape] (%)	Weight
Preceding handshape	Other	0.747	1537	59.3	0.679
	Pause	0.345	279	54.8	0.585
	1	−1.092	268	22.4	0.251
Following handshape	Pause	0.417	224	57.1	0.603
	Other	0.374	1521	59.0	0.592
	1	−0.791	339	29.5	0.312
Grammatical category & indexicality	pt:pro1	2.381	370	87.6	0.915
	pt:pro2	0.760	184	63.6	0.681
	pt:pro3	0.726	253	63.6	0.674
	pt:det	0.613	100	63.0	0.649
	Wh-signs	−0.012	178	61.2	0.497
	pt:other	−0.096	18	38.9	0.476
	pt:loc	−0.211	157	41.4	0.447
	Adverbs	−0.390	65	38.5	0.404
	pt:propl	−0.404	64	42.2	0.400
	Adjectives	−0.576	154	36.4	0.360
	Other functors	−0.659	218	33.4	0.341
	Verbs	−1.023	179	30.2	0.264
	Nouns	−1.109	144	29.9	0.248

Lexical frequency^a^	Top 5	0.791	1061	68.5	0.688
	Other	−0.791	1023	38.9	0.312

a Seperate analysis.

**Table 7:** Effect of specific preceding and following handshape variants on conditioning 1 handshape variation.

Factor group	Factor	Log odds	Tokens	Percentage of application value [other handshape] (%)	Weight
Preceding handshape	1	1.197	268	77.6	0.768
	X	1.029	22	77.3	0.737
	o	0.280	173	56.6	0.570
	6	−0.107	123	45.5	0.473
	Pauses	−0.239	279	45.2	0.440
	4	−0.432	215	39.5	0.394
	&	−0.438	38	36.8	0.392
	5	−0.518	877	37.3	0.373
	L	−0.773	89	31.5	0.316

Following handshape	1	0.974	339	70.5	0.726
	&	0.030	44	45.5	0.507
	X	−0.040	23	43.5	0.490
	4	−0.075	207	44.4	0.481
	6	−0.106	114	43.0	0.474
	o	−0.135	189	42.9	0.466
	Pauses	−0.186	224	42.9	0.454
	L	−0.198	102	41.2	0.451
	5	−0.264	842	39.2	0.434

**Table 8:** Significant and non-significant social factors conditioning variation in the 1 handshape.

Factor group	Factor	Log odds	Tokens	Percentage of application value [other handshape] (%)	Weight
Region	Cardiff	0.516	289	65.1	0.626
	Belfast	0.211	297	52.2	0.552
	Bristol	0.205	318	57.5	0.551
	London	−0.022	297	54.5	0.494
	Glasgow	−0.142	292	53.8	0.465
	Birmingham	−0.324	297	48.8	0.420
	Manchester	−0.443	294	45.9	0.391

Gender (n.s.)	Male	0.012	999	52.5	0.503
	Female	−0.012	1085	55.4	0.497

Ethnicity (n.s.)	Non-White	0.041	177	56.5	0.510
	White	−0.041	1907	53.7	0.490

Age group (n.s.)	Younger	0.003	1054	54.8	0.501
	Older	−0.003	1030	53.1	0.499

Language background (n.s.)	Deaf	0.066	671	54.2	0.517
	Hearing	−0.066	1413	53.9	0.483

Social class (n.s.)	Middle	0.018	800	54.4	0.505
	Working	−0.018	1284	53.7	0.495

**Table 9:** Significant factors conditioning variation in the 5 variant.

Factor group	Factor	Log odds	Tokens	Percentage of application value [other handshape] (%)	Weight
Preceding handshape	5	1.047	877	29.1	0.740
	Pauses	0.603	279	22.9	0.646
	L	0.166	89	15.7	0.541
	o	0.060	173	13.9	0.515
	6	0.039	123	15.4	0.510
	4	−0.014	215	13.5	0.497
	X	−0.156	22	9.1	0.461
	&	−0.543	38	13.2	0.367
	1	−1.202	268	4.9	0.231

Following handshape	5	0.829	842	29.7	0.696
	Pauses	0.573	224	21.4	0.639
	o	0.380	189	21.2	0.594
	&	0.295	44	15.9	0.573
	6	0.118	114	18.4	0.530
	4	−0.020	207	14.5	0.495
	X	−0.070	23	13.0	0.482
	1	−1.032	339	5.6	0.263
	L	−1.073	102	6.9	0.255

Grammatical function & indexicality	pt:pro1	2.036	370	48.1	0.884
	pt:pro3	0.995	253	26.1	0.730
	pt:propl	0.920	64	25.0	0.715
	pt:pro2	0.816	184	23.9	0.693
	pt:det	0.791	100	23.0	0.688
	pt:loc	0.308	157	17.2	0.576
	Wh-signs	0.083	178	15.2	0.521
	pt:other	−0.283	18	5.6	0.430
	Other functors	−0.372	218	8.7	0.408
	Verbs	−1.105	179	5.0	0.249
	Adjectives	−1.340	154	4.5	0.208
	Adverbs	−1.364	65	4.6	0.204
	Nouns	−1.486	144	3.5	0.185

Region	Cardiff	0.440	289	28.0	0.608
	Bristol	0.378	318	24.2	0.593
	Belfast	0.216	297	20.2	0.554
	London	0.102	297	21.9	0.525
	Glasgow	0.034	292	19.9	0.509
	Manchester	−0.442	294	15.6	0.391
	Birmingham	−0.728	297	12.8	0.326

**Table 10:** Significant factors conditioning variation in the L variant.

Factor group	Factor	Log odds	Tokens	Percentage of application value [other handshape] (%)	Weight
Preceding handshape	&	2.388	38	34.2	0.916
	6	2.187	123	30.9	0.899
	L	2.182	89	37.1	0.899
	5	1.338	877	16.1	0.792
	Pauses	1.323	279	17.6	0.790
	o	1.223	173	14.5	0.773
	1	0.832	268	9.7	0.697
	4	0.601	215	7.9	0.646
	X	−12.074	22	0	<0.001

Following handshape	L	0.923	102	37.3	0.716
	6	0.767	114	30.7	0.683
	Pauses	0.123	224	18.8	0.531
	5	−0.034	842	16.9	0.491
	o	−0.055	189	16.9	0.486
	X	−0.068	23	13.0	0.483
	&	−0.085	44	13.6	0.479
	1	−0.354	339	10.0	0.412
	4	−1.216	207	4.8	0.229

**Table 11:** Vertical (lateral) orientation versus other orientation.

Factor group	Factor	Log odds	Tokens	Percentage of application value [vertical orientation] (%)	Weight
Lexical item	pt:pro2	0.782	183	44.8	0.677
	pt:pro3	0.656	253	41.5	0.649
	pt:loc	−0.555	157	17.2	0.355
	pt:pro2/3pl	−0.719	53	13.2	0.319

**Table 12:** Horizontal (prone) orientation versus other orientation.

Factor group	Factor	Log odds	Tokens	Percentage of application value [horizontal orientation] (%)	Weight
Lexical item	pt:pro2/3pl	0.931	53	58.5	0.709
	pt:loc	0.581	157	49.0	0.632
	pt:pro2	−0.626	183	20.8	0.340
	pt:pro3	−0.730	253	19.8	0.317

## Footnotes

1 As is conventional in the sign linguistics literature, glosses for lexical signs are given in small caps (e.g., give). For signs requiring more than one English word, a hyphen is used (e.g., next-year). All the lexical signs in this study were assigned an ID-gloss – i.e., a unique label used to identify a specific lexeme and all its phonological and morphological variants (see Section 4.1.4 for more information about ID glossing). All the glosses used throughout this paper represent ID glosses where possible (i.e., except when discussing other studies where this approach to identifying lexical signs was not used). Pointing signs are glossed using a prefix (pt) followed by a colon and information about its function. For example, pt:pro1, pt:pro2, pt:pro3 all indicate a pointing sign that functions as a first, second, or third person singular pronoun respectively. Additional glosses used with pointing signs include pt:det, for points functioning as determiners, and pt:loc for locative points. If a pointing sign is identified as a plural point, a -pl suffix is appended to the gloss (e.g., pt:pro3pl).

2 Bayley et al. (2002) observed that the thumb-only variant of a pronominal pointing sign could be influenced by the physical location of its (second or third person) referent. As they filmed people in groups, a point (using the 1 handshape) to a referent situated to the signer’s immediate ipsilateral side (to the right for right-handed signers) may be physically uncomfortable and the thumb-only variant might be produced instead. 161 tokens were excluded from their analysis for this reason (5356 tokens originally).

3 This is in contrast to Meier’s (1990) claim that a (presumably already uniform and stable) grammatical distinction exists between first and non-first person in the ASL pronominal system.

4 For ease of comparison with Bayley et al. (2002), we have merged singular and plural pointing signs into a single category for first, second, and third person pronominals. This is why the figures in Table 4 differ from that provided in Table 3 where singular and plural points have been separated.

5 Large corpora are needed in order for lexical frequency studies to be conducted, such as those on Auslan (Johnston, 2012) and BSL (Cormier et al., 2011).

6 It is unclear whether the regional differences found in the current study (or indeed whether the range of significant social factors found in phonological variation in other studies such as Bayley et al. (2002) and Schembri et al. (2009) can be considered to be instances of ’accents’ in the sense of sociophonetic variation found in spoken languages. Foulkes and Docherty (2006) note that “the interweaving of sociophonetic and linguistic information in speech is so complete that no natural human utterance can offer linguistic information without simultaneously indexing one or more social factor” (p. 419). It is unlikely that sociophonetic information is as pervasive in signed languages but this warrants further research.
